# Ruptured Intramural Pregnancy with Myometrial Invasion Treated Conservatively

**DOI:** 10.1155/2011/965910

**Published:** 2011-11-22

**Authors:** Anis Fadhlaoui, Mohamed Khrouf, Kais Nouira, Anis Chaker, Fethi Zhioua

**Affiliations:** ^1^Department of Obstetrics and Gynecology, Aziza Othmana University Hospital (Medical University of Tunis), Place du Gouvernement, La Kasba, 1008 Tunis, Tunisia; ^2^Department of Radiology, Aziza Othmana University Hospital (Medical University of Tunis), Place du Gouvernement, La Kasba, 1008 Tunis, Tunisia

## Abstract

*Background*. Intramural pregnancy is a rare form of ectopic pregnancy, difficult to diagnose and generally complicated by uterine rupture. *Case*. A 38-year-old woman, gravida 5 para 1, was diagnosed with intramural pregnancy by ultrasound and confirmed with MRI. A uterine rupture occurred, which lead to laparotomy and a conservative treatment. *Conclusion*. Early diagnosis is necessary for conservative treatment.

## 1. Introduction

Intramural pregnancy is the rarest form of ectopic pregnancy; it is characterized by a gestation within the uterine wall, completely surrounded by myometrium and separated from the uterine cavity and the fallopian tube. It has been first reported in 1913 by Doderlein et al. [1]. So far, less than 30 cases have been reported in the literature [2]. This kind of pregnancy goes rarely further than 12 weeks' gestation. Diagnosis' establishment is difficult and is often made through surgical procedures or after extensive bleeding and uterine rupture occurring between 11 and 30 weeks' gestation which necessitates hysterectomy. The percentage of maternal mortality is approximately 2.5% [3]. It may reflect uterine trauma, adenomyosis, and invasion of the uterine wall by placenta accreta or external migration and implantation of the embryo on the serosal surface of the uterus [4–6].

We report a case of a ruptured intramural pregnancy treated conservatively.

## 2. The Case

**Figure 1:**
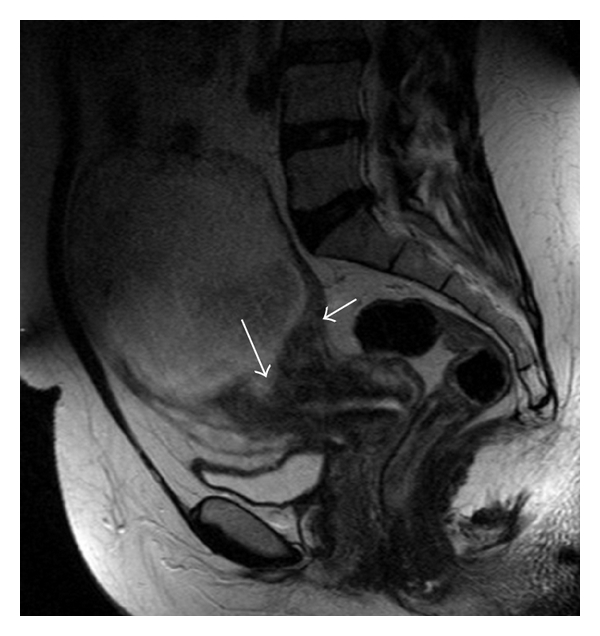
The gestational sac develops from the fundus with the presence of a sign of the spur (short arrow) and an extension of the contents of the bag in the thickness of the myometrium (long arrow). Note the exophytic development of trophoblastic tissue.

**Figure 2:**
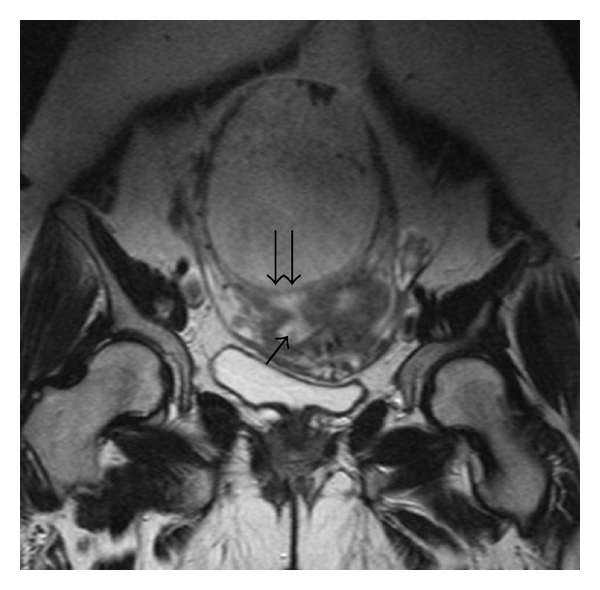
The wall of the gestational sac (double arrows) separates its contents from the uterine lumen (arrow).

A 38-year-old woman, gravida 5, para 1, abortion 2, with a history of secondary infertility and salpingectomy for an ectopic pregnancy, was referred for a suspicion of a pregnancy developing in a rudimentary cornua at 13 weeks' gestation. She was completely asymptomatic. Physical examination revealed stable vital signs while bimanual examination revealed an enlarged uterus with no adnexal masses. Transvaginal ultrasound revealed a gestational sac with a heartbeating fetus along with measurements corresponding to 13 weeks' gestation. The endometrial cavity was quite near in the right cornual region, but there was no evident communication between them. Both the endometrium and gestational sac appeared to be surrounded by myometrium. The diagnosis of intramural pregnancy was suspected and a Magnetic Resonance Imaging (MRI) was indicated. It revealed a gestational sac with a fetus developing inside the fundic uterine wall (Figure 1). The placenta seemed to invade the myometrium (Figure 2).

A uterine artery embolization (UAE) was selected in order to reduce bleeding during laparotomy. While preparing the patient for embolization, she developed signs suggesting an acute abdomen (diffuse distention and tenderness and rebound tenderness on abdominal examination) and a hypovolemic shock. An emergency laparotomy was performed. About 300 mL of blood and clot was detected, the uterus was ruptured, and the placenta was actually percreta but did not invade any abdominal organ. The fetus was still in the gestational sac. Enucleation and removal of the gestational sac then padding of the residual myometrial cavity were performed. Trophoblastic tissue was removed easily, and we did not need to perform any myometrial resection or subtotal hysterectomy. She had a satisfactory recovery. 

## 3. Discussion

Intramural pregnancy refers to a conceptus implanting within the myometrium without connection with the fallopian tubes and endometrial cavity [7]. It is a rare type of ectopic pregnancy in which hysterectomy is most often unavoidable, because of extensive bleeding and uterine rupture occurring between 11 and 30 weeks' gestation [2, 3]. Intramural pregnancy can have various etiologies. It may reflect uterine trauma (previous curettage, cesarean section, myomectomy, etc.) [4, 5], microscopic sinus tracts associated with adenomyosis, invasion of the uterine wall by placenta accreta and subsequent growth of the fetus deep within the myometrium, or external migration and implantation of the ovum on the serosal surface of the uterus [1, 6]. According to Lu et al. [1], adenomyosis seems to be the most reasonable factor in the development of intramural pregnancy. This is due to the fact that deep adenomyosis has enough endometrial tissue to respond to estrogen and progesterone and demonstrate decidualization, which could be a potential site for blastocyst implantation [1, 7]. Several cases of intramural pregnancy secondary to embryo transfer in IVF (in vitro fertilization) procedures had been reported [2]. In our patient's case, two risk factors could be involved, uterine trauma (D&C and salpingectomy) and the placenta percreta, since increased trophoblastic activity and defective decidualization let the conceptus penetrate into the myometrium (the trophoblastic tissue could then continue to invade the uterine wall to become percreta).

Abdominal pain and metrorrhagia in the presence of a positive pregnancy test are the hallmark signs of ectopic pregnancy. However, early diagnosis may not be easy, and ultrasound may not easily localize the gestation. Usually, the diagnosis of intramural pregnancy is not made until uterine rupture, and surgery is performed [2, 8]. The typical ultrasound appearance of intramural pregnancy is a gestational sac completely surrounded by myometrium [8] as described in our case. Up until 2005, there were only 3 cases of intramural pregnancy that have been correctly diagnosed preoperatively by ultrasound [1, 2, 9]. The ultrasound appearance can mimic a degenerating myoma or a pregnancy in a sacculation, in a diverticulum, or in a congenital uterine anomaly [6, 10, 11]. In our case, a separate gestational sac with a positive heart beat fetus was individualized in the myometrium away from the endometrial cavity by ultrasound. This was also verified by MRI. Gestational sac within the dark myometrium is seen as bright signal intensity surrounded by a thin mantle of myometrium [12]. Laparoscopic surgery may be necessary to differentiate between intramural pregnancy and any another ectopic pregnancy (tubal or cornual).

The intramural pregnancy treatment depends on the stage in which it is diagnosed. With uterine rupture and hypovolemic shock, emergency hysterectomy is often necessary. If intramural pregnancy is discovered prior to rupture, conservative or expectant management could be considered, including surgical enucleation, systemic, or local methotrexate injection [6], and fertility can be maintained. Successful expectant management was reported for the first time by Bernstein et al. [2]. Systemic or local injection of methotrexate has been successfully used for the management of intramural pregnancy. Low doses of methotrexate are preferable to avoid uterine rupture after active bleeding into the myometrium [13]. The use of systemic or local methotrexate in our case did not seem appropriate because of the late gestational age with a heartbeating fetus. To our knowledge no case of UAE in such an indication has been reported before.

The diagnosis of intramural pregnancy in the early gestational age is of a great importance, because it allows medical or conservative surgical treatment, thus, preventing severe morbidities, hysterectomy, and sparing fertility.

## 4. Conclusion

Intramural pregnancy is the rarest form of ectopic pregnancy. The association of transvaginal ultrasound and MRI provides important information for the diagnosis by achieving the delineations of gestational sac within the myometrium. Early diagnosis is necessary for a conservative treatment.
